# GM-CSF-loaded chitosan hydrogel as an immunoadjuvant enhances antigen-specific immune responses with reduced toxicity

**DOI:** 10.1186/s12865-014-0048-x

**Published:** 2014-10-18

**Authors:** Kyung Hee Noh, Yeong Min Park, Hyuk Soon Kim, Tae Heung Kang, Kwon-Ho Song, Young-Ho Lee, Yeongseon Byeon, Hat Nim Jeon, In Duk Jung, Byung Cheol Shin, Kyung-Mi Lee, Seung-Yong Seong, Hee Dong Han, Tae Woo Kim

**Affiliations:** Laboratory of Infection and Immunology, Graduate School of Medicine, Korea University, Gojan-1 Dong, Ansan-Si, Gyeonggi-Do 425-707 South Korea; Department of Biochemistry, College of Medicine, Korea University, Seoul, Korea; Department of Immunology, School of Medicine, Konkuk University, 268 Chungwondaero, Chungju-Si, Chungcheongbuk-Do 380-701 South Korea; Department of Immunology and Physiology and Functional Genomics Institute, College of Medicine, Konkuk University, 268 Chungwondaero, Chungju-Si, Chungcheongbuk-Do 380-701 South Korea; Research Center for Medicinal Chemistry, Division of Drug Discovery Research, Korea Research Institute of Chemical Technology, 141 Gajeong-ro, Yuseong-gu Daejeon, 305-600 South Korea; Department of Microbiology and Immunology, College of Medicine, Seoul National University, 28 Yongon-dong, Jongno-gu Seoul, 110-799 Republic of Korea; Department of Biomedical Sciences, College of Medicine, Seoul National University, 28 Yongon-dong, Jongno-gu Seoul, 110-799 Republic of Korea

**Keywords:** Adjuvant, Chitosan, Hydrogel, Immune response

## Abstract

**Background:**

The application of vaccine adjuvants has been vigorously studied for a diverse range of diseases in order to improve immune responses and reduce toxicity. However, most adjuvants have limited uses in clinical practice due to their toxicity.

**Methods:**

Therefore, to reduce health risks associated with the use of such adjuvants, we developed an advanced non-toxic adjuvant utilizing biodegradable chitosan hydrogel (CH-HG) containing ovalbumin (OVA) and granulocyte-macrophage colony-stimulating factor (GM-CSF) as a local antigen delivery system.

**Results:**

After subcutaneous injection into mice, OVA/GM-CSF-loaded CH-HG demonstrated improved safety and enhanced OVA-specific antibody production compared to oil-based adjuvants such as Complete Freund’s adjuvant (CFA) or Incomplete Freund’s adjuvant (IFA). Moreover, CH-HG system-mediated immune responses was characterized by increased number of OVA-specific CD4^+^ and CD8^+^ INF-γ^+^ T cells, leading to enhanced humoral and cellular immunity.

**Conclusions:**

In this study, the improved safety and enhanced immune response characteristics of our novel adjuvant system suggest the possibility of the extended use of adjuvants in clinical practice with reduced apprehension about toxic side effects.

**Electronic supplementary material:**

The online version of this article (doi:10.1186/s12865-014-0048-x) contains supplementary material, which is available to authorized users.

## Background

Vaccine adjuvants have been extensively studied in order to enhance their safety and improve the immune responses elicited by the accompanying antigen for immunotherapy [1-3]. However, conventional adjuvants evoke serious side effects due their toxicity [4]. This has limited their use in clinical trials in recent years and poses as a serious hurdle to effective adjuvant-based immunotherapy, resulting in side effects such as boil and pyrexia occurring in clinical trials. Mineral oil-based media, Complete Freund’s adjuvant (CFA) and Incomplete Freund’s adjuvant (IFA) [5,6], are available as commercialized products and are potent adjuvants commonly used in animal research, as they can augment both humoral and cellular immune responses to a wide range of antigens. However, they are toxic to human subjects, and their use in animals are now also discouraged or banned by many institutional animal ethics committees due to their noxious side effects [4]. These limitations enforce the need for novel and improved adjuvant systems to replace the existing ones.

Here, we have developed an improved chitosan hydrogel (CH-HG) system as a vaccine adjuvant to enhance induced immune responses and reduce toxicity [7,8]. Chitosan is particularly attractive for clinical and biological applications due to its low toxicity, low immunogenicity, biocompatibility, and biodegradability [8,9]. In addition, CH-HG displays a liquid-solid phase transition depending on temperature, allowing CH-HG to simply be injected at the diseased site without a requirement for surgical procedure. Therefore, CH-HG may lead to enhanced safety and immune responses at disease sites. Moreover, CH-HG can be gradually degraded by enzymes in the body after the antigen has been completely released.

Granulocyte-macrophage colony-stimulating factor (GM-CSF) has been used as a vaccine adjuvant, which stimulates macrophage differentiation and proliferation, and to activate antigen presenting cells (APCs) such as dendritic cells (DCs) or macrophages. These qualities make GM-CSF important and relevant to developing vaccine therapies [10-12]. GM-CSF can augment the primary immune response of murine spleen cells to sheep red blood cells and increase interleukin-1 secretion [13]. In addition, GM-CSF can increase the immunogenicity of tumors in animal models and stimulate both humoral and cellular immune responses, thus serving as an effective adjuvant for DNA or peptide based vaccines [14,15]. This increased immune response is presumed to be a result of the GM-CSF-mediated increase in maturation and function of APCs [16]. Further studies, using microspheres of encapsulated GM-CSF mixed with irradiated tumor cells that were injected subcutaneously also demonstrated increased tumor immunogenicity [17]. These studies motivated us to ask whether GM-CSF function could be improved in prolonged immune responses at a local adjuvant site. Here, we present a novel immunization strategy using CH-HG-loaded GM-CSF/OVA as an adjuvant system to increase immunogenicity in both humoral and cellular immune responses in a mouse model as a proof-of-concept, and in order to approach a clinically relevant study of GM-CSF in CH-HG as an enhanced adjuvant for vaccination that evokes an improved immune response, while exhibiting lower toxicity compared to existing adjuvants.

## Methods

### Mice

Female C57BL/6 mice (6 weeks old, 20 g) were purchased from Daehan Biolink (Chungbuk, Korea) and maintained under a protocol approved by the Korea University Institutional Animal Care and Use Committee (KUIACUC-2013-210). All procedures were performed in accordance with recommendations for the proper animal care and use.

### Preparation of OVA and GM-CSF-loaded CH-HG

We have previously reported the physical characteristics of CH-HG as an *in vitro* or *in vivo* depot system after subcutaneous (SC) injection [8,9]. We prepared CH-HG containing OVA and/or GM-CSF with mild modification made to our previously reported method. Briefly, chitosan solution (medium molecular weight of 161 kDa, viscosity of 200,000 cps and a degree of deacetylation of 80%) was obtained by dissolving 40 mg of chitosan in 1.8 ml of 0.1 M HCl solution. Glycerol 2-phosphate disodium salt hydrate (β-GP) solution containing 50 μg of OVA and/or 50 ng of GM-CSF was prepared by dissolving 0.2 g of β-GP and predetermined amount of OVA or GM-CSF in 0.2 ml of distilled water. The CH solution was cooled to 4°C and continuously stirred while adding 0.2 ml of mixed solution was added. The final product is successfully formed hydrogel at body temperature and physiological pH *in vivo* after subcutaneous (SC) injection into mice. Notably, the mice did not suffer severe side effects, including pus formation or inflammation, and maintained a healthy appearance after CH-HG implantation.

### Safety test of CH-HG in mice after SC injection

To confirm the safety of adjuvants, 50 μl of CH-HG, Complete Freund’s adjuvant (CFA), or Incomplete Freund’s adjuvant (IFA) were injected subcutaneously into mice. The external morphologies of the adjuvants were monitored in the mice, and the hydrogel volume was measured using calipers for 14 days. After 14 days from initial administration, we measured body weights to evaluate the toxicity of each adjuvant.

### Immune response against CH-HG containing OVA + GM-CSF

Fifty microliters of CH-HG, CFA, and IFA containing OVA + GM-CSF were injected subcutaneously into mice, and the adjuvants were boosted at 7 days using the same injection volume. The IgG, IgG1, and IgG2a levels in serum were measured by ELISA 3 weeks after the first immunization. Briefly, the presence of OVA-specific antibodies in the sera from CH-HG mediated immunization of C57BL/6 mice (five per group) was determined by ELISA using microwell plates coated with OVA protein. Purified OVA protein was diluted to 0.5 μg/ml in PBS buffer (pH 7.4), and 50 μl of that solution was then added to each well of 96-well microtiter plates. Purified OVA protein was used as a negative control. The plates were incubated overnight at 37°C, followed by three washes with 300 μl of PBS. After washing, 200 μl of blocking solution (1% skim milk) was incubated at 37°C for 1 hr, and then the plates were washed three additional times using PBS containing 0.05% Tween20 (PBS-T). Serial dilutions of the tested sera were made (0.1 ml/well), and the plates were incubated for 1 hr at 37°C. The plates were washed with PBS-T and incubated with 0.1 ml of alkaline phosphatase-conjugated rabbit anti-mouse antibodies (Zymed) per well for 1 hr at 37°C. The plates were again washed with PBS-T and incubated with alkaline phosphatase substrate (100 μl/well, Sigma) according to the manufacturer’s instructions for 1 hr at 37°C. Plates were read on a MicroElisa reader at a wavelength of 650 nm (Additional file 1).

### Flow cytometry analysis

To assess OVA specific immune responses, we immunized mice with 50 μl of one of five different solutions; 1) 50 μg of OVA solution, 2) 50 μg of OVA solution with GM-CSF, 3) CH-HG containing 50 μg of OVA, 4) CH-HG containing 50 μg of OVA +50 ng of GM-CSF, or 5) IFA containing 50 μg of OVA +50 ng of GM-CSF. Splenocytes were harvested from the immunized mice (five per group) for 2 weeks after the first immunization. Prior to intracellular cytokine staining, 5 × 10^6^ pooled splenocytes from each immunization group were incubated overnight with 1 μg/ml OVA peptide containing MHC class I epitope (aa 257-264) or MHC class II epitope (aa 323-339) to detect OVA-specific CD4^+^ and CD8^+^ T cell precursors. Intracellular IFN-γ and IL4 staining, as well as flow cytometric analysis were performed using a Becton-Dickinson FACScan with CELLQuest software (Becton Dickinson Immunocytometry Systems, Mountain View, CA). The number of OVA-specific INF-γ or IL4 secreting CD4^+^ T cells and INF-γ secreting CD8^+^ T cells were assessed by intracellular cytokine staining and FACScan analysis.

### Statistical analysis

Differences in continuous variables were analyzed by Student’s *t*-test for comparing two groups and ANOVA was performed to compare differences between multiple groups. For values that were not normally distributed, the Mann-Whitney rank sum test was used. The statistical package for the Social Sciences (SPSS, Inc.) was used for all statistical analyses. A *p* value of <0.05 was considered statistically significant.

## Results

### CH-HG safety after SC injection in mice

**Figure 1:**
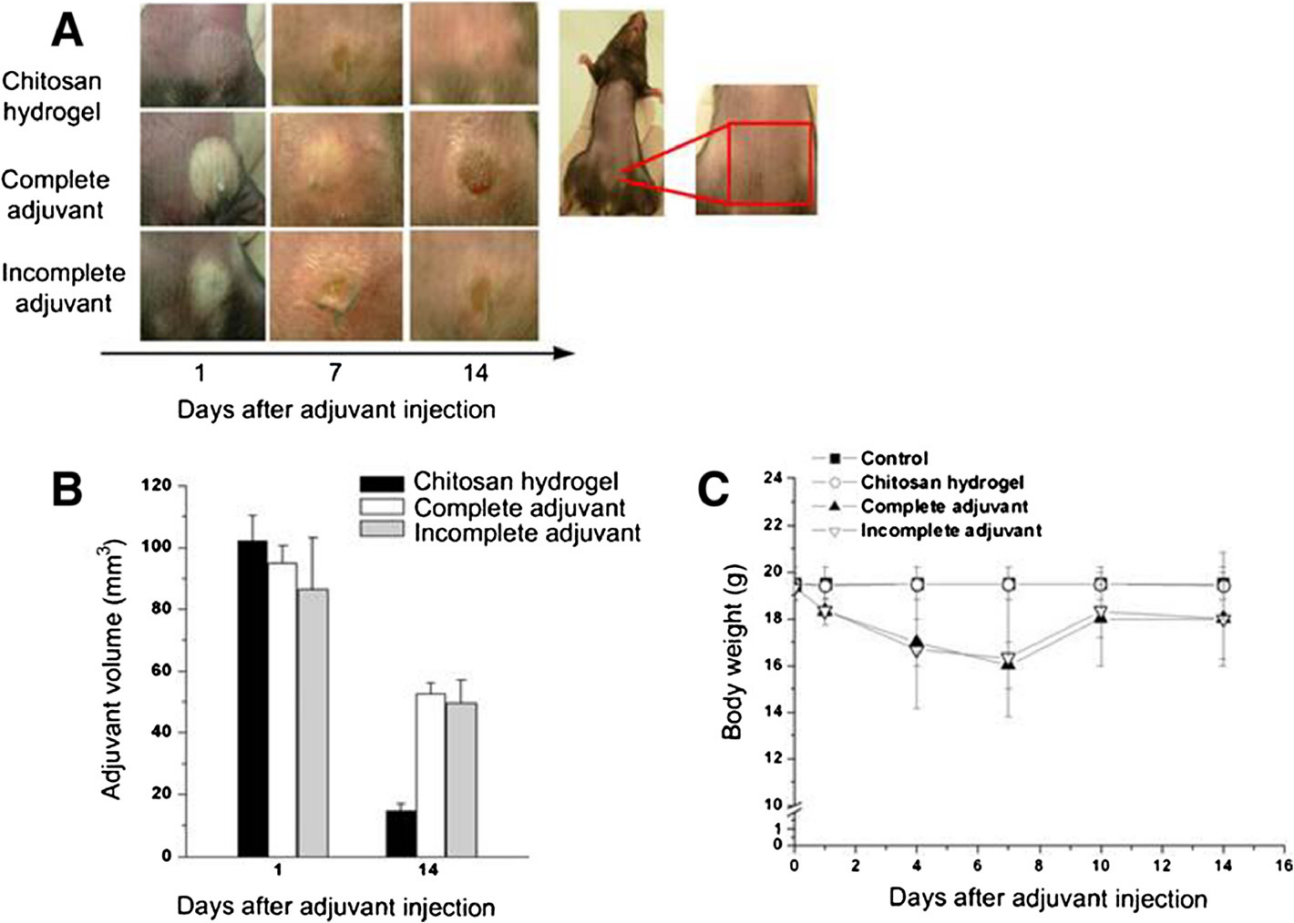
**Safety of CH-HG compared to that of commercial adjuvants, in mice after subcutaneous administration. (A)** Morphology of the injection site in mice. **(B)** Change in volume of injected adjuvants after 14 days. **(C)** Body weight of mice. The data are presented as the means ± S.D. (n = 5).

We first confirmed hydrogel formation after SC injection of CH into mice (Figure 1A). The CH solution displayed a liquid-solid phase transition in a temperature-dependent manner and formed an endothermic hydrogel at the injection site. Furthermore, we noted no pus, discharge, or scab formation at the injection site of CH-HG solutions, indicating improved safety compared to commercially available CFA or IFA (Figure 1A). Moreover, CH-HG was not present at the injection site 2 weeks after injection, suggesting increased biodegradation compared to CFA and IFA (Figure 1B). We also checked the body weights of mice for 2 weeks after SC injection of CH-HG to evaluate adjuvant toxicity (Figure 1C). While CFA and IFA injected groups weighted less, the CH-HG injected group did not weigh significantly different than the control group, suggesting that the CH-HG system displayed enhanced safety at the in injection site. Therefore, CH-HG may be useful as a safer adjuvant system in mice.

### CH-HG containing OVA and GM-CSF induced titers of OVA specific IgG

**Figure 2:**
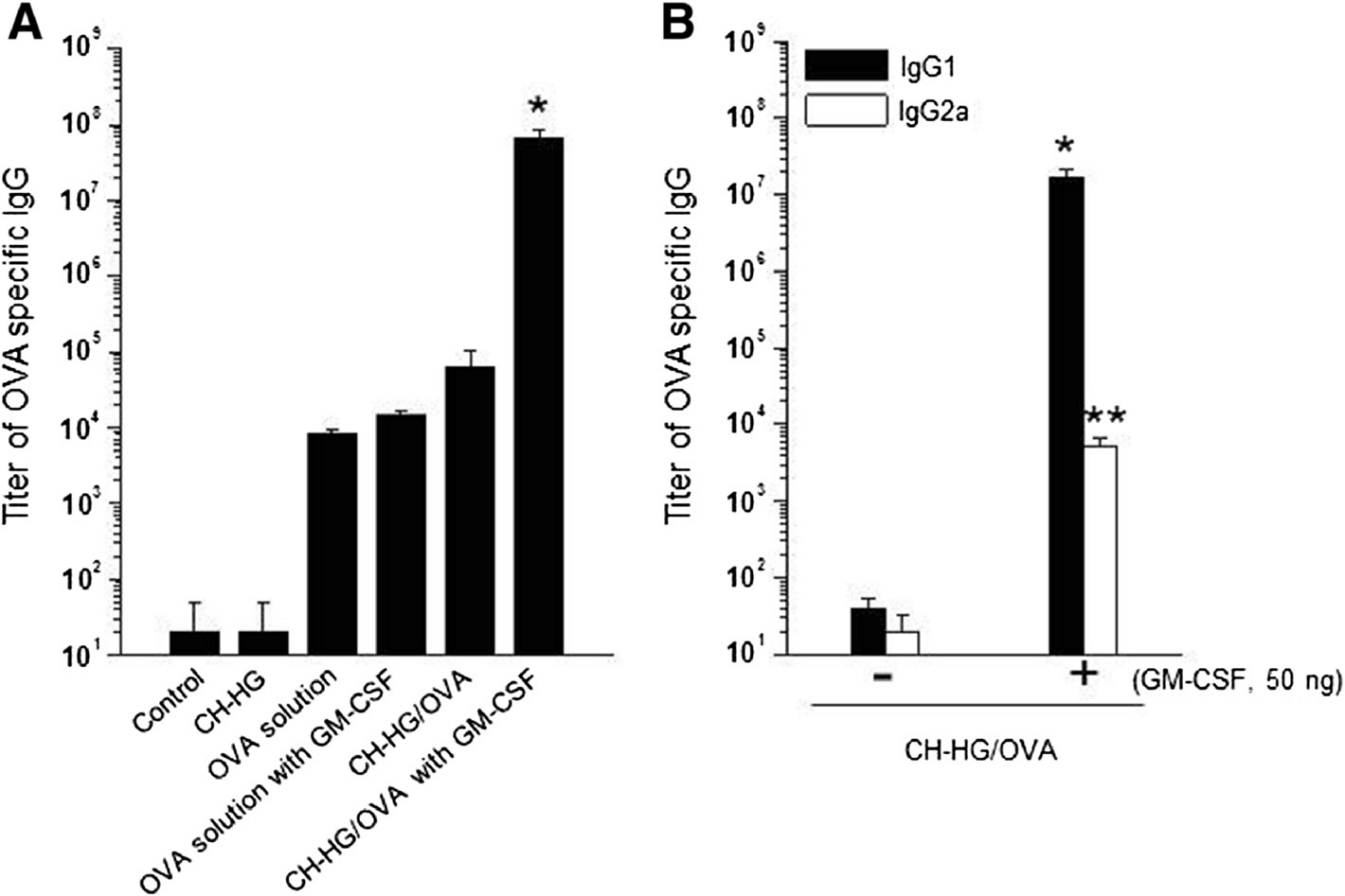
**OVA-specific immune responses in mice immunized with 50 μg of OVA solution, CH-HG containing 50 μg of OVA, and CH-HG containing 50 μg of OVA and 50 ng of GM-CSF.** Sera were collected from immunized mice (3 per group) 2 weeks after the last immunization and were used to characterize OVA-specific antibodies. Mice received an immunization with 50 μg of OVA per mouse via subcutaneous injection. Seven days after immunization, experimental groups were boosted. **(A)** Titers of OVA specific IgG. **(B)** Titers of OVA specific IgG for humoral or cellular immune response. The data are presented as the means ± S.D. (n = 5), (*p <0.001, (**p <0.002).

To determine the effectiveness of CH-HG in inducing an immune response, we assessed the humoral immune response to CH-HG containing OVA (CH-HG/OVA). Two weeks after the last SC injection of CH-HG/OVA into mice, OVA specific IgG expression was significantly induced, and measured 10-fold higher than that in mice immunized with soluble OVA alone (*p* <0.001) (Figure 2A). Additionally, OVA specific IgG progressively increased rising to a 10^7^-fold increase in expression 5 weeks after the last immunization (Additional file 2: Figure S1). Based on the immune response data for CH-HG/OVA, we next tested immune responses for CH-HG/OVA with 50 ng of GM-CSF (CH-HG/OVA + GM-CSF), which further increased the IgG titer (10^3^-fold increase) compared to the CH-HG/OVA without GM-CSF (*p* <0.001). We therefore chose CH-HG/OVA + GM-CSF for all subsequent experiments (Figure 2A). In addition, there were higher titers of OVA-specific IgG1 than of IgG2a in serum from mice immunized with CH-HG/OVA + GM-CSF (Figure 2B). These data demonstrate that immunization with CH-HG/OVA + GM-CSF significantly enhances OVA-specific humoral immune response (IgG1), and the CH-HG induced immune response would be strongly dependent on the Th2 type immune response.

### CH-HG/OVA + GM-CSF induction of OVA-specific IgG compared to CFA and IFA

**Figure 3:**
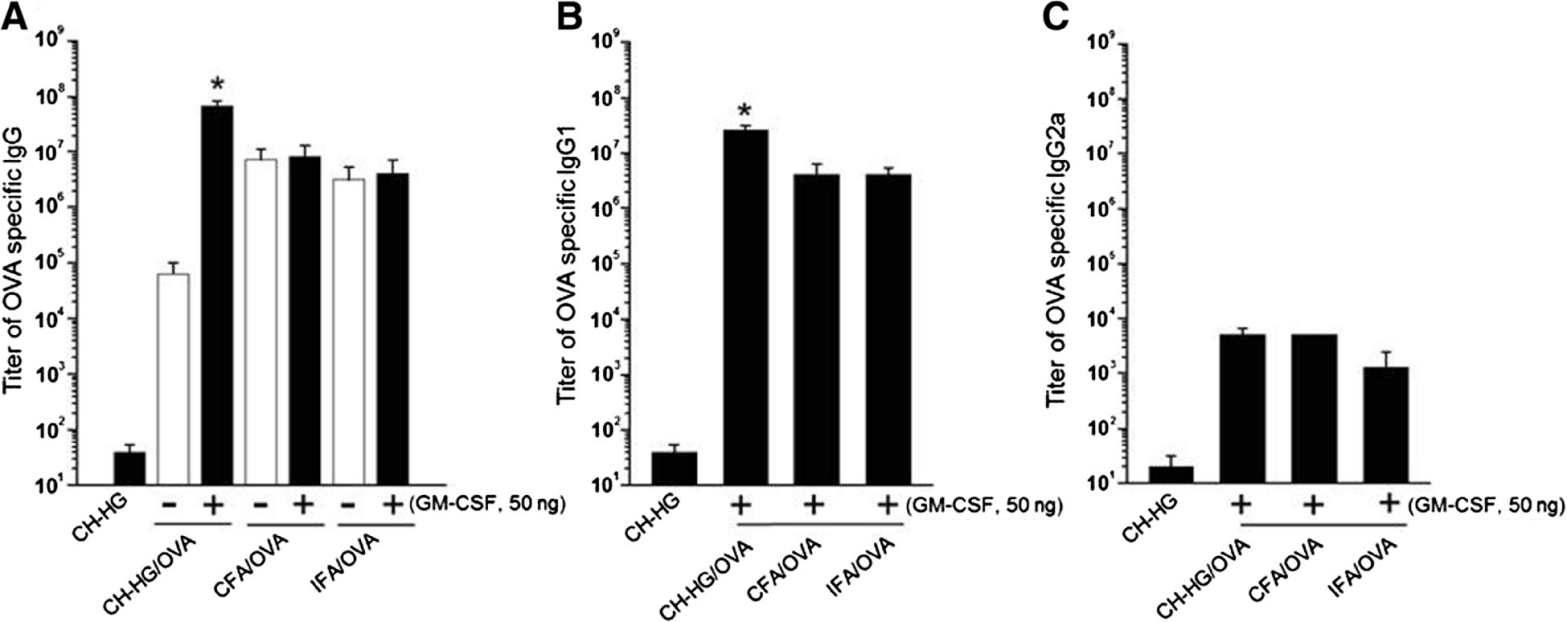
**OVA-specific immune responses in mice immunized with various adjuvants with or without GM-CSF.** Sera were collected from adjuvant-immunized mice (5 per group) 2 weeks after the last immunization and were used to characterize OVA-specific antibodies. Mice received an immunization with 50 μg of OVA and 50 ng of GM-CSF via subcutaneous injection. Seven days after immunization, experimental groups were boosted. **(A)** Titers of OVA-specific IgG in mice immunized with various adjuvants. **(B)** Titers of OVA-specific IgG1 in mice immunized with various adjuvants. **(C)** Titers of OVA-specific IgG2a in mice immunized with various adjuvants. The data are presented as means ± S.D. (n = 5), (*p <0.01).

Next, we checked whether CH-HG could increase OVA specific IgG expression compared to commercialized CFA or IFA. We first measured OVA specific IgG from the serum of mice immunized with CH-HG/OVA + GM-CSF, CFA/OVA + GM-CSF, or IFA/OVA + GM-CSF (Figure 3A). Mice immunized with CH-HG/OVA + GM-CSF generated significantly higher titers of OVA-specific antibody responses compared to mice immunized with CFA/OVA + GM-CSF or IFA/OVA + GM-CSF (*p* <0.01). Furthermore, the sera of mice immunized with CH-HG/OVA + GM-CSF had significantly higher titers of OVA-specific IgG1 antibodies compared to mice immunized with CFA/OVA + GM-CSF or IFA/OVA + GM-CSF (Figure 3B). These data indicate that immunization with CH-HG/OVA + GM-CSF elicits a stronger OVA-specific humoral immune response, providing a potential alternative in clinical practice to existing commercially available adjuvants such as CFA or IFA. However, the IgG2a titers were similar between all groups of mice (Figure 3C), which suggests that CH-HG/OVA + GM-CSF may not contribute the IgG2 mediated immune responses.

### CH-HG/OVA + GM-CSF generated an enhanced OVA-specific cellular immune response

**Figure 4:**
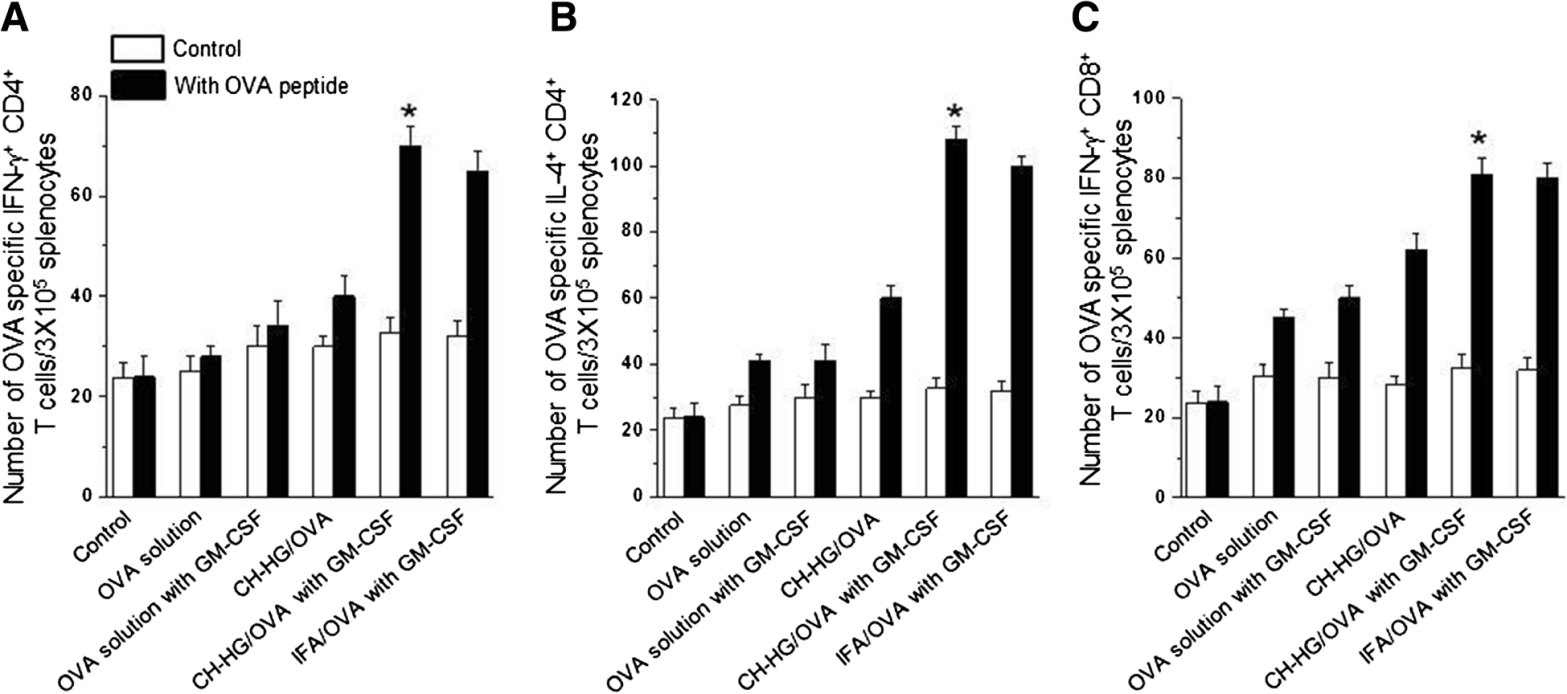
**CH-HG/OVA + GM-CSF immunization generated enhanced OVA-specific CD4** ^**+**^ **and CD8** ^**+**^ **T cell immune responses compared to control or CH-HG/OVA.** C57BL/6 mice (5 per group) were immunized with CH-OVA/GM-CSF. **(A)** OVA-specific CD4^+^ T cell immune responses were assessed in the immunized mice. **(B)** The bar graph depicts the number of OVA-specific IL4-secreting CD4^+^ T cells per 3 × 10^5^ splenocytes. **(C)** The bar graph depicts the number of IFN-γ producing OVA-specific CD8^+^ T cell precursors per 3 × 10^5^ splenocytes from mice after immunization with OVA, CH-HG/OVA or CH-HG/OVA + GM-CSF. The data are presented as means ± S.D. (n = 5), (*p <0.001).

T cell mediated immunity is important for controlling tumor cells and intracellular infections caused by bacteria or viruses. For a quantitative characterization of OVA-specific CD4^+^ T cell-mediated immune responses, we performed flow cytometry analysis with or without the MHC class II-restricted peptide OVA_323-339_ (ISQAVHAAHAEINEAGR). As shown in Figure 4A and 4B, the numbers of both OVA-specific Th1 CD4^+^ and Th2 CD4^+^ T cells in the splenocytes of CH-HG/OVA + GM-CSF immunized mice were significantly increased compared to that of mice immunized with OVA or CH-HG/OVA (*p* <0.001). We further measured CD8^+^ T cell immune responses by performing intracellular cytokine staining with or without MHC class I-restricted peptide OVA_257-264_ (SIINFEKL). As shown in Figure 4C, the numbers of OVA-specific CD8^+^ T cells in splenocytes of mice immunized with CH-HG/OVA + GM-CSF were higher than those of the other groups (*p* <0.001). Collectively, these data demonstrate that immunization with CH-HG/OVA + GM-CSF adjuvant could elicit antigen-specific cellular immune responses.

## Discussion

Adjuvant-based vaccination in immunotherapy is highly effective, but it is associated with inherent safety and toxicity problems that need to be overcome before use in clinical settings [3,4]. We demonstrate here that a novel CH-HG based adjuvant system loaded with GM-CSF, a broad immune-modulating cytokine, led to potent both humoral and cellular immune responses without severe side effects. This approach has broad utility for adjuvant mediated therapeutic materials as well as other immunotherapy applications.

Several synthetic biomaterials have been proposed as adjuvants for effective antigen delivery. These include microparticles [18], nanofibers [19], metal-based particles [20], and emulsions [21]. Although many types of compounds can be potentially used as delivery agents, their safety and evoked immune responses cause concerns. The development of adjuvant-based vaccination for immunotherapy, therefore, requires clinically suitable, safe, and effective delivery systems.

Therapeutic materials packaged into CH-HG may provide a clinically viable approach for the development of a vaccine related immunotherapy. The biocompatibility and biodegradability of these systems are key parameters for medical and pharmaceutical applications [9]. Here, we demonstrate that a novel hydrogel-based adjuvant system loaded with OVA and GM-CSF led to potent immune responses in mice. This study implements a novel adjuvant with improved safety and reduced toxicity. In addition, the CH-HG system allows for the co-delivery of therapeutic materials, such as an antigen, which could further enhance the antigen specific immune response without increasing toxicity. Injection of CH-HG is simple because CH-HG is a liquid phase at room temperature and therefore, does not require surgery for insertion at the site of the disease, which would disrupt the disease environment. Moreover, the biodegradation rate of injected hydrogel can be controlled by modifying the injected volume. In this study, we injected 50 μl of chitosan solution, which degrades in approximately 2 weeks, suggesting that this amount can be used for repeated injections. This local depot system may be attractive for many biomedical applications, including administration of anesthetic agents after surgery and treatment of certain skin, breast, or neck cancers. Such an approach may also be useful for adjuvant therapy, or as a local treatment for chronic periodontitis. Although the CH-HG mediated adjuvant platform can be useful for diseases associated with the immune system and with the intent to enhance immune response, additional possibilities of optimized loading for effective cytokine or immune modulator using the CH-HG platform may be explored and developed for research purposes.

## Conclusion

In summary, we have developed a novel adjuvant system that yields high immune responses without negative side effects at the local site of interest due to matrix toxicity. Our data demonstrate that CH-HG based local antigen delivery can invoke antigen specific CD8^+^ T cell immunity. Such CH-HG based adjuvant strategies may have broad potential as antigen delivery platforms in human disease and represent an opportunity for further development of vaccine based immunotherapeutics.

## Additional files

Additional file 1
**Arrive guidelines checklist.**
Additional file 2: Figure S1 OVA-specific humoral immune responses in mice immunized with CH-HG/OVA + GM-CSF over time. Sera were collected from CH-HG/OVA + GM-CSF-immunized mice (5 per group) for 2 weeks after the last immunization and were used to characterize OVA-specific antibodies. Mice received an immunization with 50 μg of OVA and 50 ng of GM-CSF per mouse via subcutaneous injection. The data are presented as means ± S.D. (n = 5).
