# Supplementary figures and images for: GM-CSF-loaded chitosan hydrogel as an immunoadjuvant enhances antigen-specific immune responses with reduced toxicity
Source: BMC Immunol. 2014 Oct 18;15:48. doi: 10.1186/s12865-014-0048-x (PMC4201920; doi:10.1186/s12865-014-0048-x)

**Additional file 1: Figure S1**


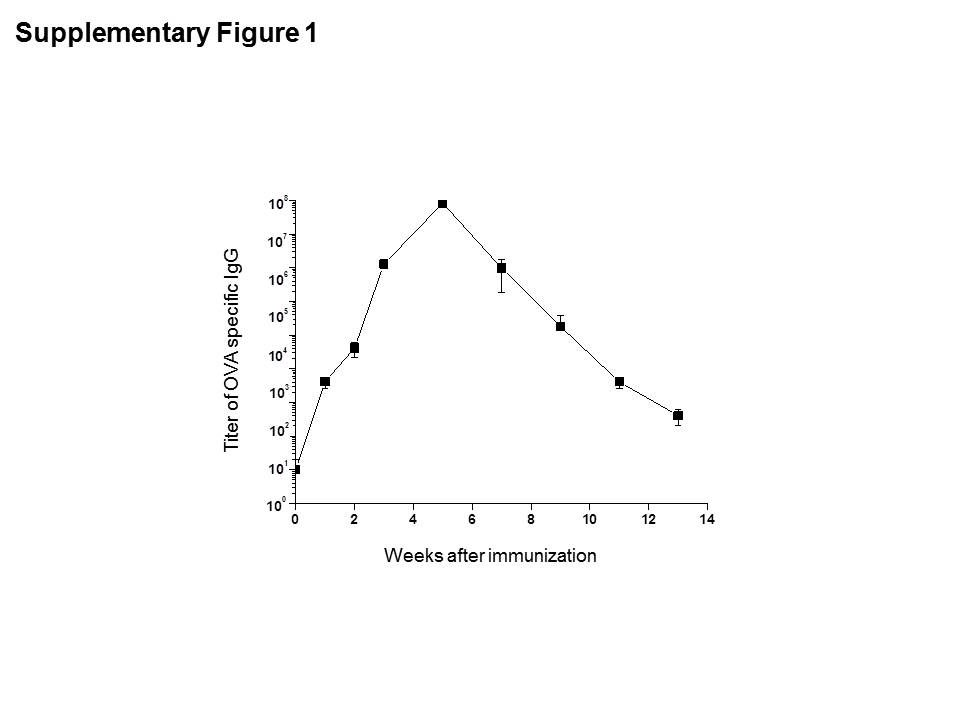

Supplement: Additional file 1 — Arrive guidelines checklist. [file 12865_2014_48_MOESM1_ESM.docx]
